# Livestock abundance predicts vampire bat demography, immune profiles and bacterial infection risk

**DOI:** 10.1098/rstb.2017.0089

**Published:** 2018-03-12

**Authors:** Daniel J. Becker, Gábor Á. Czirják, Dmitriy V. Volokhov, Alexandra B. Bentz, Jorge E. Carrera, Melinda S. Camus, Kristen J. Navara, Vladimir E. Chizhikov, M. Brock Fenton, Nancy B. Simmons, Sergio E. Recuenco, Amy T. Gilbert, Sonia Altizer, Daniel G. Streicker

**Affiliations:** 1 Odum School of Ecology, University of Georgia, Athens, GA 30602, USA; 2 Center for the Ecology of Infectious Disease, University of Georgia, Athens, GA 30602, USA; 3 Department of Microbiology and Immunology, Montana State University, Bozeman, MT, USA; 4 Department of Wildlife Diseases, Leibniz Institute for Zoo and Wildlife Research, Berlin, Germany; 5 Center for Biologics Evaluation & Research, U.S. Food & Drug Administration, Rockville, MD, USA; 6 Department of Poultry Science, University of Georgia, Athens, GA, USA; 7 Department of Biology, Indiana University, Bloomington, IN, USA; 8 Facultad de Ciencias, Universidad Nacional de Piura, Piura, Perú; 9 Programa de Conservación de Murciélagos de Perú, Piura, Perú; 10 Department of Pathology, College of Veterinary Medicine, University of Georgia, Athens, GA, USA; 11 Department of Biology, Western University, London, Ontario, Canada; 12 Department of Mammalogy, Division of Vertebrate Zoology, American Museum of Natural History, New York, NY, USA; 13 Department of Preventive Medicine and Public Health, Faculty of Medicine, Universidad Nacional Mayor de San Marcos, Lima, Perú; 14 National Wildlife Research Center, United States Department of Agriculture, Fort Collins, CO, USA; 15 Institute of Biodiversity, Animal Health and Comparative Medicine, University of Glasgow, Glasgow, UK; 16 MRC–University of Glasgow Centre for Virus Research, Glasgow, UK

**Keywords:** agriculture, *Bartonella*, ecoimmunology, haemoplasmas, resource provisioning, supplemental feeding

## Abstract

Human activities create novel food resources that can alter wildlife–pathogen interactions. If resources amplify or dampen, pathogen transmission probably depends on both host ecology and pathogen biology, but studies that measure responses to provisioning across both scales are rare. We tested these relationships with a 4-year study of 369 common vampire bats across 10 sites in Peru and Belize that differ in the abundance of livestock, an important anthropogenic food source. We quantified innate and adaptive immunity from bats and assessed infection with two common bacteria. We predicted that abundant livestock could reduce starvation and foraging effort, allowing for greater investments in immunity. Bats from high-livestock sites had higher microbicidal activity and proportions of neutrophils but lower immunoglobulin G and proportions of lymphocytes, suggesting more investment in innate relative to adaptive immunity and either greater chronic stress or pathogen exposure. This relationship was most pronounced in reproductive bats, which were also more common in high-livestock sites, suggesting feedbacks between demographic correlates of provisioning and immunity. Infection with both *Bartonella* and haemoplasmas were correlated with similar immune profiles, and both pathogens tended to be less prevalent in high-livestock sites, although effects were weaker for haemoplasmas. These differing responses to provisioning might therefore reflect distinct transmission processes. Predicting how provisioning alters host–pathogen interactions requires considering how both within-host processes and transmission modes respond to resource shifts.

This article is part of the theme issue ‘Anthropogenic resource subsidies and host–parasite dynamics in wildlife’.

## 1. Introduction

Human activities such as agriculture, urbanization and recreational feeding of wildlife can create abundant, predictable food resources for many species [1]. While supplemental resources can benefit wildlife facing seasonal food shortages, they can also alter pathogen transmission in ways that have negative consequences for human and animal health [2,3]. Resource provisioning can create novel assemblages of host species around anthropogenic resources that can enable pathogen spillover. For example, increased spatial overlap between mango plantations and pig farms in Malaysia have attracted flying foxes to abundant fruit, facilitating the cross-species transmission of Nipah virus from bats to pigs and humans [4]. Provisioning can also increase infection by altering host demographic and behavioural processes, such as increasing fecundity and aggregation [5], which can amplify pathogen transmission through density dependence and increased contact with infectious stages [6–8]. However, provisioning sometimes has the opposite effect of reducing infection. For example, red foxes in Switzerland foraging on urban waste were less frequently infected with a zoonotic tapeworm compared to rural foxes [9]. Declining pathogen transmission associated with provisioning could occur if improved nutrition enhances host resistance to or recovery from infection [10–12]. As immune defences are energetically costly [13], supplemental feeding can alleviate trade-offs between immunity and other processes (e.g. growth rate [14]) or between different arms of the immune system [15]. Provisioning can also improve immunity by reducing starvation stress; ad libitum access to food increased antibody production in deer mice [16] and allowed voles to mount stronger defences against nematodes [17]. As a final level of complexity, pathogens in the same host may have opposite responses to provisioning owing to differences in transmission modes or interactions with the immune system [2,18]. Although predicting when provisioning can increase or decrease infection in wildlife is important to manage disease risks [19,20], few studies have explored cross-scale links between food availability, immunity and infection in natural systems.

The common vampire bat (*Desmodus rotundus*) has experienced major ecological changes from provisioning throughout its range in Latin America [21]. Although uncommon to rare in undisturbed habitats [22], vampire bats are abundant in agricultural landscapes [23]. While vampire bats historically fed on wild mammals in forested habitats, populations residing near humans now preferentially feed on livestock and poultry [24,25]. Access to these prey types increases bat feeding success [23,26], which could improve bat immune defence owing to their physiological sensitivity to starvation [27,28]. Bats occupying livestock-dense habitat could thus show lower physiological stress and improved immune measures. However, livestock-dense habitat could also suppress bat immunity and increase infection through other mechanisms [29]. For example, increases in bat density from greater reproductive success or immigration [23,30] could increase chronic stress (compromising immunity) and contribute to a large susceptible pool that increases infection risk and shifts allocation of immune defence [31,32]. Thus, changes in pathogen transmission from provisioning could reflect either direct effects of feeding on livestock on individual bat immunity or indirect effects of occupancy in agricultural habitats.

Here, we conducted a 4-year field study of vampire bats across 10 sites in Peru and Belize that differ in livestock abundance to investigate how resource provisioning predicts changes in host demography, immunity and infection. To test the prediction that provisioned bats shift foraging towards livestock prey, we first assessed relationships between livestock abundance and bat feeding patterns as revealed by isotopic analysis of bat hair samples. Second, to test the prediction that greater availability of livestock stimulates bat demographic processes, we examined associations between livestock abundance and two measures of bat demography: reproductive status and sex. The latter represents an ecologically relevant measure in this system because higher frequencies of males in provisioned sites could reflect biased sex ratios from improved maternal condition [30,33] or more immigration of males to food-dense habitats [30,34]. Third, we assessed the relative importance of diet (inferred from isotope analyses) and local livestock abundance for eight measures of bat immunity, including humoral and cellular effectors of innate and adaptive immunity [35]. We lastly tested if and how provisioning-mediated variation in immunity was linked to infections with two intracellular bacteria common in bats: *Bartonella* spp. and haemotropic *Mycoplasma* spp. (i.e. haemoplasmas) [36,37]. While their transmission routes in bats are poorly understood, *Bartonella* is generally spread by arthropod vectors [38,39], while haemoplasmas transmit from direct contact (i.e. through blood and saliva) and potentially from vector-borne exposure [40–42]. Host immune responses to these pathogens could also differ; for example, *Bartonella* often produces asymptomatic infection in reservoir hosts [43], while haemoplasma pathology can range from asymptomatic to acute and chronic anaemia [41]. Differential responses of these bacterial infections to provisioning could therefore reflect contrasting transmission modes or different immune defences. We used statistical tools for assessing hypothesized causal relationships to assess the potential for effects of provisioning on infection to be mediated through observed immunological variation.

## 2. Material and methods

### (a) Field sites and livestock abundance

**Figure 1.**
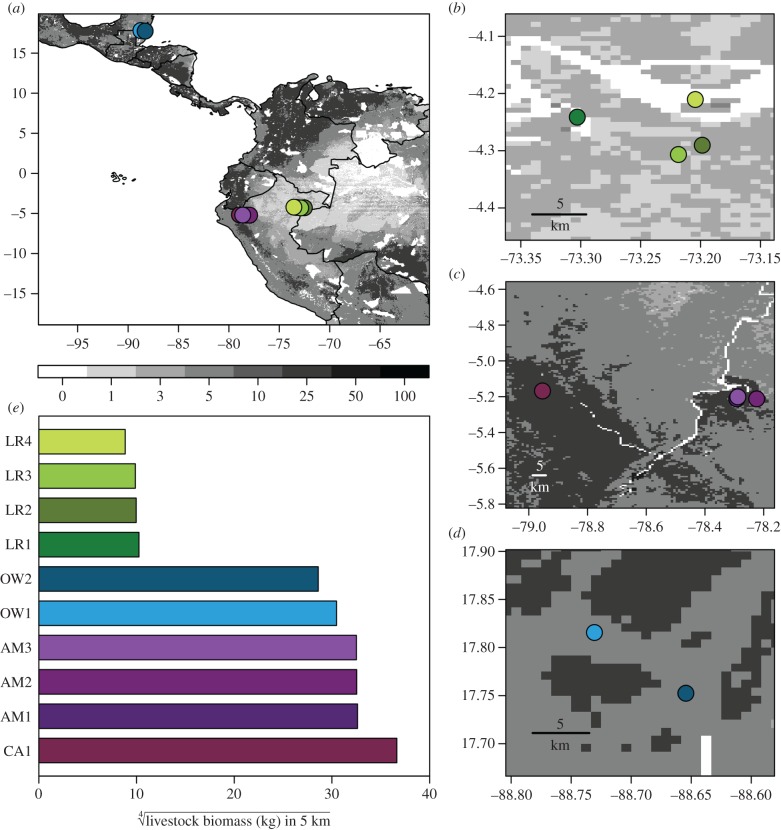
Vampire bat sampling sites in Peru and Belize (*a*), where shading and colour strip represent the log biomass (kilogram) of cows, pigs, and chickens from the GLW and AnAge databases [44,45]. Fine-scale patterns in livestock biomass are shown in (*b*) Loreto, (*c*) Amazonas and Cajamarca and (*d*) Belize; site coordinates are jittered to reduce overlap. (*e*) Quarter-root-transformed livestock biomass within 5 km of each capture location. Colours correspond to sampling region: green, Loreto; purple, Amazonas and Cajamarca; blue, Belize.

Between July 2013 and September 2016, we sampled 369 vampire bats across 10 sites in Peru (Departments of Cajamarca, Amazonas and Loreto) and Belize (Orange Walk District; figure 1*a*). Sampling consisted of capture–recapture over 2–5 nights per site. In 2013–2014, we sampled regions in distinct years (Amazonas and Cajamarca in 2013, Belize and Loreto in 2014). All sites were sampled 1–2 times annually in 2015–2016, although sampling did not occur across all seasons for all sites owing to logistical constraints (e.g. Loreto was mostly sampled in summer). Sites consisted of broadleaf deciduous, upland or flooded forest and varied in their agricultural intensity. Sites in Peru included intact forest and areas with small- to intermediate-scale cattle farming (figure 1*b*,*c*) [46,47], while sites in Belize were located within a matrix of agricultural habitat (figure 1*d*) [48]. Four capture sites were structures (trees, caves, cistern and Mayan ruins) known to be inhabited by vampire bats. Other sites (*n* = 6) included capture near livestock corrals or chicken coops where bat bites had been recently reported.

We quantified livestock abundance as the total biomass of mammalian livestock (cattle, pigs) and poultry (chickens) (hereafter livestock biomass) within a 5 km radius of each site using the 2014 Gridded Livestock of the World (GLW) database of modelled livestock abundance estimates [44] and average species mass (kilogram) from the AnAge Database [45]. GLW data were provided at a 1 km resolution and were processed and assigned to sites (figure 1*e*; electronic supplementary material, table S1) using the *raster* package in R [49]. Livestock biomass was quarter-root transformed and binned into regions of low and high-livestock abundance owing to a nearly binary distribution (figure 1*e*); however, results of our analyses were similar when livestock biomass was treated as continuous.

### (b) Bat capture and sampling

Vampire bats were captured in mist nets or harp traps placed at roost exits, along flight paths or outside livestock corrals from 19.00 to 05.00. Upon capture, bats were placed in individual holding bags and issued a uniquely coded Incoloy wing band (3.5 mm, Porzana Inc.). We classified age as juvenile, sub-adult or adult based on fusion of phalangeal epiphyses [47,48]. Reproductive activity was indicated by the presence of scrotal testes in males and by the evidence of pregnancy or lactation in females. For isotopic analysis of diet, we trimmed less than 5 mg hair from the back of each bat. To quantify bat immune measures, we obtained up to 150 µl blood by lancing the propatagial vein with a sterile 23-gauge needle, followed by collection with heparinized capillary tubes. Thin blood smears were prepared on glass slides and stained with buffered Wright–Giemsa (Camco Quik Stain II). Plasma was obtained by centrifuging blood in serum separator tubes and was stored on cold packs until freezing at –20°C and long-term storage at –80°C. Up to 30 µl blood was stored on Whatman FTA cards to preserve bacterial DNA. Except for 14 bats that were humanely sacrificed for other studies, all bats were released at their capture site.

### (c) Stable isotope analysis

Stable carbon (^13^C) and nitrogen (^15^N) isotope signatures were determined from dried bat hair samples using a Thermo Delta V isotope ratio mass spectrometer at the University of Georgia Center for Applied Isotope Studies. Isotope values were expressed in standard *δ* notation, where *δ*^13^C or *δ*^15^N = [(*R*_sample_/*R*_standard_) − 1] × 1000, and *R* is the ratio of ^13^C/^12^C or ^15^N/^14^N. Analyses used two standards per 12 samples for *δ*^13^C and *δ*^15^N: bovine (*σ* = 0.05, 0.30 and *μ* = –21.75, 7.44) or 1577c (*σ* = 0.08, 0.10 and *μ* = –17.52, 8.12) and spinach (*σ* = 0.23, 0.42 and *μ* = –27.39, –0.48).

Vampire bat feeding on livestock has been differentiated from feeding on wildlife using *δ*^13^C, as most grasses consumed by livestock use the C4 pathway and most forest plants consumed by wildlife use the C3 pathway [24,50]. *δ*^15^N also provides inference into trophic level, as consumer *δ*^15^N is enriched by 3–4‰ relative to its diet [51]. We opportunistically collected samples from known prey species in each study region to quantify differences in bat feeding patterns while accounting for different geographical isotopic baselines [23,25,48,52,53]. Prey included cattle (*Bos* spp.), horses (*Equus caballus*), chickens (*Gallus domesticus*), pigs (*Sus scrofa domesticus*), goats (*Capra aegagrus hircus*), tapir (*Tapirus bairdii*), red brocket (*Mazama americana*) and white-tailed deer (*Odocoileus virginianus*), peccaries (*Tayassu* spp.) and lowland paca (*Cuniculus paca*); individual prey *δ*^13^C and *δ*^15^N are presented in electronic supplementary material, figure S1 and table S2. For each study region, we calculated the minimum distance in isotopic space between each bat and any mammalian livestock and poultry to estimate consumption of provisioned food [54]. We did not use mixing models as prey coverage was uneven between regions.

### (d) Quantifying bat immune components

We used leucocyte profiles from blood smears to measure investment in cellular immunity [55] and chronic stress, given that high ratios of neutrophils to lymphocytes can indicate elevated blood glucocorticoid hormones [56]. We estimated total white blood cells (WBCs) as the average number of leucocytes from 10 fields of view at 400× magnification with light microscopy [57]; quantitative counts (e.g. with the Unopette system) were not performed owing to limited blood volumes and remote field sites. Nucleated cell differentials recorded the percentage of neutrophils, lymphocytes, monocytes, eosinophils and basophils by counting 100 leucocytes at 1000× magnification. Total WBC estimates were normalized with a quarter-root transformation.

We assessed humoral innate immunity by quantifying the *ex vivo* bacterial killing ability (BKA) of plasma against *Escherichia coli* ATCC 8739 [58], which is mediated mostly through complement proteins [59]. We used the microplate reader method [60], using 1:8 dilutions of plasma to phosphate-buffered saline (PBS) run in 22 µl duplicates and challenged with 5 µl of a 10^4^ bacteria/ml solution in PBS (E power Microorganisms no. 0483E7, Microbiologics Inc.) [48]. To quantify humoral adaptive immunity, we measured immunoglobulin G (IgG) antibody in plasma with a protein G ELISA [61], which binds IgG from many wildlife taxa including bats [62]. We diluted 3 µl of each sample to 1 : 30 000 with 50 mM NaHCO_3_ buffer (pH 9.5) and ran 100 µl of each sample in duplicate using protein G–horseradish peroxidase conjugate (P21041, Life Technologies) [48]. We included human IgG (MP Biomedicals, LLC) as a positive control. As antibody concentration is proportional to optical density (OD), we analysed the mean IgG OD.

### (e) Pathogen detection

Blood smears were screened for extracellular haemoparasites (trypanosomes and microfilariae) by microscopically reviewing 100 fields of view at 400× magnification [63]. For detection of bacteraemia, genomic DNA was isolated from blood on Whatman FTA cards using QIAamp DNA Investigator Kits (Qiagen). For *Bartonella* spp.*,* we used nested PCR to amplify a region of the citrate synthase gene (*gltA*), which has high discriminatory power for differentiating among *Bartonella* [64], using previously published primers [65]. For haemoplasmas, we amplified the partial 16S rRNA haemoplasma gene using previously published primers [42,66].

### (f) Statistical analysis

We first used generalized linear mixed models (GLMMs) fitted with restricted maximum-likelihood (REML) and Gaussian errors with *lme4* to test if bat *δ*^13^C and *δ*^15^N varied across study regions; bat identification number (ID) was nested within site as a random effect to account for repeat sampling of individuals (*n* = 16) and similar values within sampling locations [67]. To test if livestock biomass predicted bat diet, we used a permutation multivariate analysis of variance (PERMANOVA) to relate livestock biomass to bat isotopic position (matrix of *δ*^13^C and *δ*^15^N; *n* = 304) and fitted another GLMM to correlate livestock biomass and the minimum isotopic distance of bats from livestock and poultry prey. To test if livestock biomass predicted bat reproduction (*n* = 362) and sex (*n* = 364), we next fitted GLMMs with binomial errors, a logit link and the same random effect structure. For all models, we calculated marginal *r^2^* 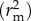 and conditional *r^2^* 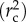 to assess fit [68] and used Moran's *I* to assess spatial autocorrelation in model residuals [69]. Year was also included as a categorical covariate in all models to control for inter-annual variation.

To analyse immunological data, we used principal component analysis (PCA) to collapse eight measures (electronic supplementary material, table S2; |*ρ*| ranged from 0.01 to 0.98, |*μ*| = 0.17) into one axis [70]. The PCA included the proportion of each WBC type, estimated WBCs, BKA and IgG, with variables centred and scaled to have unit variance (*n* = 160; electronic supplementary material, figure S2). PC1 accounted for 30% of the variance and was loaded positively by neutrophils (0.61), BKA (0.24), estimated WBCs (0.10) and basophils (less than 0.01), and negatively by lymphocytes (–0.59), monocytes (–0.32), eosinophils (–0.26) and IgG (–0.21). As neutrophils, BKA and total WBCs are markers of innate immunity and inflammation, while lymphocytes and IgG are metrics of adaptive immunity [35], we interpret larger PC1 values as more investment in innate immunity and less investment in adaptive immunity. Negative loading by monocytes in particular suggest our PCA does not fully divide along a functional innate–adaptive axis, as these leucocytes are typically categorized as part of innate immunity. However, monocytes can also play key roles in initiating an adaptive immune response by their differentiation into macrophages and dendritic cells [71].

We tested relationships between provisioning and bat immunity with a PERMANOVA that evaluated how all immune measures correlate with livestock biomass and bat diet while controlling for year. To assess the relative contribution of livestock biomass and bat diet on immunity, we used maximum-likelihood to fit GLMMs with PC1 as the response variable, bat ID nested in site as a random effect, and livestock biomass, minimum isotopic distance from livestock and poultry, year, bat age, sex and reproductive status as fixed effects with appropriate interactions (electronic supplementary material, table S3). We generated a candidate set of all additive GLMMs, limited to a maximum of four covariates each to keep the number of models low (*R* = 86) relative to our sample excluding missing values (*n* = 151) [72]. We compared GLMMs with the Akaike information criterion corrected for small sample size (AICc) and refitted models with REML to calculate 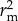 and 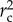. We used model averaging to estimate mean effect sizes and 95% confidence intervals for how all fixed effects correlate with the immunity PC1. Averaging was performed across LMMs whose cumulative Akaike weight (*w_i_*) summed to 95%, and mean coefficients were standardized with partial standard deviation [73]. We used *MuMIn* and *lme4* for model averaging [74,75].

To understand the relationships between both provisioning covariates and bat immunity on bacterial infection, we fitted univariate GLMMs with binomial errors, a logit link and bat ID nested in site as a random effect separately for infection with *Bartonella* and haemoplasmas; we adjusted for multiple comparisons with the Benjamini–Hochberg correction [76]. We next used causal mediation analysis (CMA) to test support for theorized causal relationships between provisioning, bat immunity and infection status. CMA estimates how much of a direct relationship between two variables (i.e. outcome model) is mediated indirectly through a third variable (i.e. mediator model) [77]. The mediator model was given as a GLMM for the immune PC1 with livestock biomass and isotopic distance from livestock as predictors. For the outcome models, we fitted two GLMMs with both provisioning covariates and the immunity PC1 to reduced datasets (*n* = 119 for *Bartonella* and *n* = 116 for haemoplasmas) to accommodate missing values. We performed CMA with 5000 Monte Carlo draws using the *mediation* package to estimate the proportion of the relationship between provisioning covariates and infection mediated through the immunity PC1 [78]; only bat ID was included as a random effect in GLMMs for the CMA owing to repeated measures and as the *mediation* package cannot support multilevel models.

## 3. Results

### (a) Livestock biomass, bat diet and demography

Bat feeding strategies were highly variable across sites (electronic supplementary material, figure S1). Bats in Loreto, where livestock biomass was generally lower, had lower *δ*^13^C (*X*^2^ = 16.22, *p* < 0.001) and higher *δ*^15^N (*X*^2^ = 48.74, *p* < 0.001) than bats in Amazonas, Cajamarca and Belize, where the livestock biomass was greater. PERMANOVA confirmed livestock biomass predicted *δ*^13^C and *δ*^15^N, explaining 52% of the variation in bat isotopic space after controlling for sampling year (*F*_1,299_ = 333.84, *p* < 0.001). Comparison of isotopes from bats and prey suggested bats in low-livestock sites (e.g. LR3) foraged mostly on poultry and wildlife, while bats in high-livestock sites (e.g. CA1) fed mostly on livestock and poultry. As most bats probably fed on some form of domestic prey, minimum isotopic distance from livestock and poultry did not vary with livestock biomass (electronic supplementary material, figure S3; *X*^2^ = 2.08, *p* = 0.15, 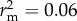). After controlling for sampling year, bats in low-livestock sites had isotopic signatures as closely aligned to these prey as did bats within high-livestock sites.

**Figure 2.**
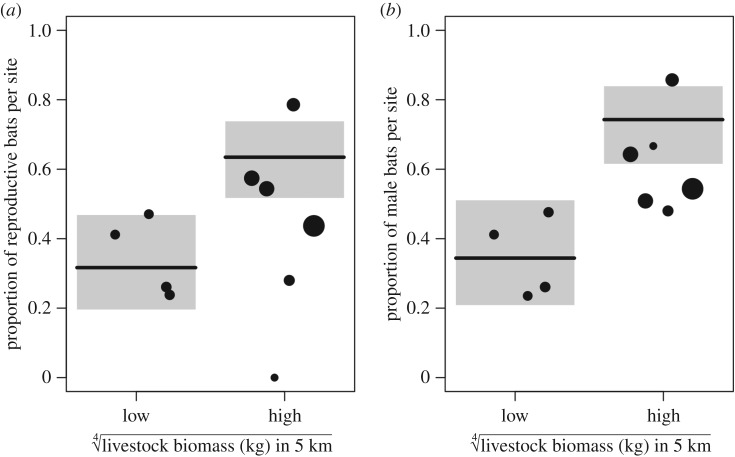
Relationships between livestock biomass and vampire bat demography. Livestock biomass predicts increases in the proportions of (*a*) reproductive and (*b*) male bats. Lines and grey shading display the fit and 95% confidence intervals from GLMMs controlling for year. Overlaid are proportion of reproductive and male bats per site, with size scaled by sample size.

Bat demography showed a stronger relationship with livestock biomass. After controlling for sampling year, livestock biomass was positively associated with the proportion of reproductive bats per site (*X*^2^ = 14.65, *p* < 0.001, 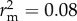; figure 2*a*) and the proportion of male bats per site (*X*^2^ = 17.82, *p* < 0.001, 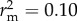; figure 2*b*). Isotopic and demographic models showed no residual spatial autocorrelation (Moran's *I* < 0.01, *p* = 0.17–0.76).

### (b) Immunological correlates of provisioning

**Figure 3.**
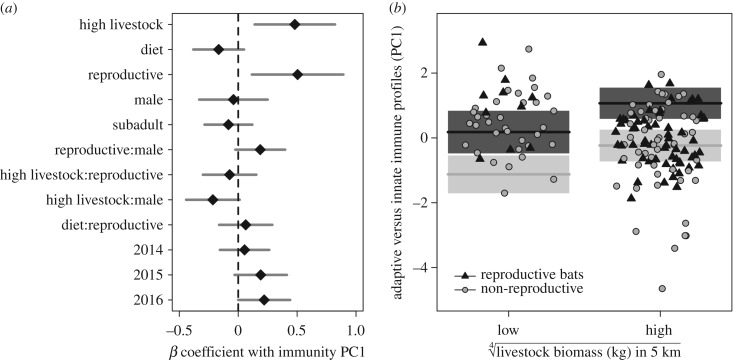
Predictors of bat immune profiles (PC1); PC1 loads positively with innate immunity and negatively with adaptive immunity. (*a*) Model averaging results across the 95% confidence set of GLMMs, with 95% confidence intervals shown in grey and mean coefficients shown by black diamonds. The dashed line represents no correlation between covariates and immunity (*β* = 0). (*b*) Results from the top GLMM; points, model fit and 95% confidence intervals are shaped and coloured by bat reproduction.

Measures of provisioning predicted differences in individual bat immunity; livestock biomass explained 9% of the variation in immune profiles (PERMANOVA; *F*_1,149_ = 16.39, *p* < 0.001), while isotopic distance from livestock explained 4% of this variation (*F*_1,149_ = 8.38, *p* < 0.001). When we applied averaging across the 95% confidence set of GLMMs (figure 3*a*; electronic supplementary material, table S3), immunity PC1 values positively correlated with livestock biomass (*β* = 0.48, 95% CI = 0.14–0.82) but showed no relationship with isotopic distance from provisioned food (*β* = –0.17, 95% CI = –0.38 to 0.05). Accounting for log time between capture and blood sampling (*n* = 127; 5–713 min) only narrowed the confidence interval for the relationships between immunity and livestock biomass (*β* = 0.46, 95% CI = 0.25–0.68) but did not affect the relationships with diet (*β* = –0.10, 95% CI = –0.31 to 0.12; electronic supplementary material, figure S4A). We obtained similar results when restricting this only to bats held for under four hours (*n* = 115; electronic supplementary material, figure S4B). Stronger effects of livestock biomass in comparison to bat diet were also reflected in this covariate having greater relative importance (0.97) than isotopic distance (0.11); reproduction, sex, year and age had relative importance of 1.00, 0.33, 0.20 and 0.16, respectively, though the mean coefficients for sex, age and bats from 2014 did not depart from zero (figure 2*a*); bats from 2015 and 2016 had increasingly higher PC1. Competitive GLMMs (ΔAICc ≤ 2) contained livestock biomass, reproductive status, age, sex and isotopic distance (table 1), and the top model was the most parsimonious, containing livestock biomass and reproduction (ΔAICc = 0.00, *w_i_* = 0.13, 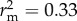). This GLMM identified immune PC1 values to be greatest in high-livestock sites (*X*^2^ = 5.65, *p* = 0.02) and for reproductive bats (*X*^2^ *=* 37.42, *p* < 0.001; figure 3*b*). These GLMMs showed no residual spatial autocorrelation (table 1).

**Table 1.** 95% confidence set of GLMMs predicting the immunity PC1. GLMMs are ranked by ΔAICc with renormalized Akaike weights (*w_i_*), number of estimated coefficients (*k*), marginal and conditional *r^2^* statistics, and Moran's *I* and *p*-value from tests of spatial autocorrelation on model residuals. A random effect of bat ID nested within site is included in all GLMMs.

immunity PC1 ∼ fixed effects	*k*	ΔAICc	*w_i_*	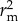	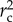	*I*	*p-*value
livestock + reproduction	3	0.00	0.13	0.33	0.39	0.007	0.53
livestock * sex + reproduction	5	0.48	0.10	0.34	0.41	0.007	0.52
isotope distance + livestock + reproduction	4	0.78	0.09	0.32	0.42	0.007	0.55
livestock + reproduction * sex	5	0.88	0.08	0.34	0.41	0.005	0.60
livestock + reproduction + year	6	1.33	0.07	0.38	0.43	0.005	0.61
isotope distance + livestock + reproduction + year	7	1.46	0.06	0.38	0.48	0.005	0.60
age + livestock + reproduction	4	1.54	0.06	0.33	0.4	0.007	0.55
livestock + reproduction + sex	4	1.75	0.05	0.33	0.4	0.007	0.55
livestock * reproduction	4	1.83	0.05	0.33	0.4	0.008	0.51
isotope distance * reproduction + livestock	5	2.57	0.04	0.32	0.42	0.006	0.57
age + isotope distance + livestock + reproduction	5	2.57	0.04	0.32	0.42	0.007	0.55
isotope distance + livestock * reproduction	5	2.62	0.03	0.32	0.42	0.007	0.53
isotope distance + livestock + reproduction + sex	5	2.76	0.03	0.32	0.42	0.007	0.55
age + livestock + reproduction + year	7	2.96	0.03	0.38	0.44	0.004	0.62
livestock + reproduction + sex + year	7	3.09	0.03	0.38	0.44	0.004	0.63
livestock * reproduction + year	7	3.18	0.03	0.38	0.43	0.006	0.57
age + livestock * reproduction	5	3.34	0.02	0.33	0.40	0.007	0.52
age + livestock + reproduction + sex	5	3.45	0.02	0.33	0.40	0.006	0.55
livestock * reproduction + sex	5	3.62	0.02	0.33	0.40	0.007	0.52
reproduction	2	4.72	0.01	0.20	0.35	0.012	0.41

### (c) Links between provisioning, immunity and bacterial infection

Prevalence of *Bartonella* and haemoplasmas in 173 bats as assessed by PCR was 70% and 68%, ranging from 40 to 100% for *Bartonella* and 45–86% for haemoplasmas by site; neither bacteria were detected microscopically. Coinfection prevalence was 54% (95% CI = 0.46–0.61; *n* = 169) and infection was positively associated; bats positive for *Bartonella* had higher odds of infection with haemoplasmas (odds ratio = 3.66, *p* < 0.01). Among 290 bats for which we screened microscopically for haemoparasites, we detected no trypanosomes and only one microfilariae from a bat in AM3.

**Figure 4.**
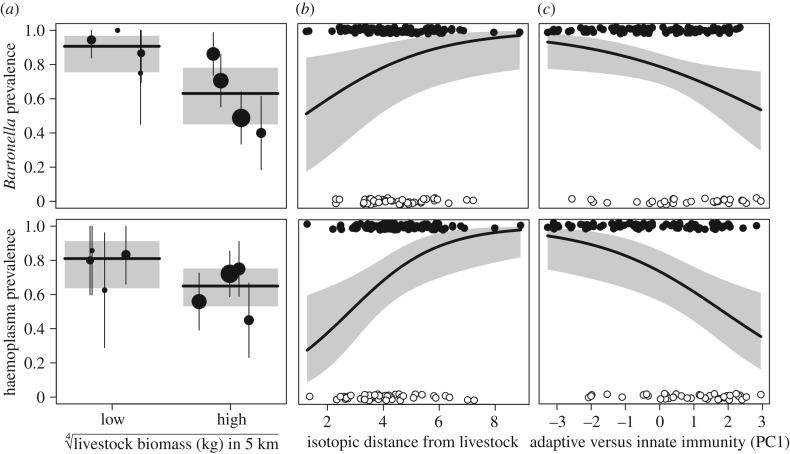
Univariate relationships between provisioning, bat immunity and bacterial infection. Modelled relationships between livestock biomass (*a*), minimum isotopic distance to livestock (mammalian and poultry, *b*), and immune profiles (immune PC1, *c*) and individual infection with *Bartonella* (top) and haemoplasmas (bottom). GLMM predictions are overlaid with 95% confidence intervals in grey and either infection prevalence and 95% confidence intervals per site (for livestock biomass) or individual infection status (jittered for isotopes and immunity).

GLMMs showed that the odds of both infections tended to decline with livestock biomass (figure 4*a*), though effect size for *Bartonella* was stronger and significant (OR = 0.18, *p* = 0.02) compared to that for haemoplasmas (OR = 0.43, *p* = 0.07). Infection with both bacteria was related to individual bat feeding patterns (figure 4*b*), with prevalence greater for bats feeding less frequently on livestock or poultry (*Bartonella*: OR = 1.57, *p* = 0.02; haemoplasmas: OR = 1.89, *p <* 0.01). Bat immunity was also associated with infection status, with lower odds of infection for bats investing more in innate immunity and less in adaptive immunity (figure 4*c*). This effect size was stronger for haemoplasmas (OR = 0.57 *p* < 0.01) than for *Bartonella* (OR = 0.67, *p* = 0.02). CMA showed that while 25% of the relationship between livestock biomass and *Bartonella* was mediated through the association between livestock biomass and bat immunity (*p* = 0.12), more substantial mediation was detected with livestock biomass for haemoplasmas (49%, *p* = 0.05). By contrast, no mediation was observed for *Bartonella* (7%, *p* = 0.74) or haemoplasmas (6%, *p* = 0.29) for the relationship between individual bat diet, immunity and infection status. No models showed significant residual spatial autocorrelation (|Moran's *I*| = 0.03–0.04, *p* = 0.06–0.58).

## 4. Discussion

**Figure 5.**
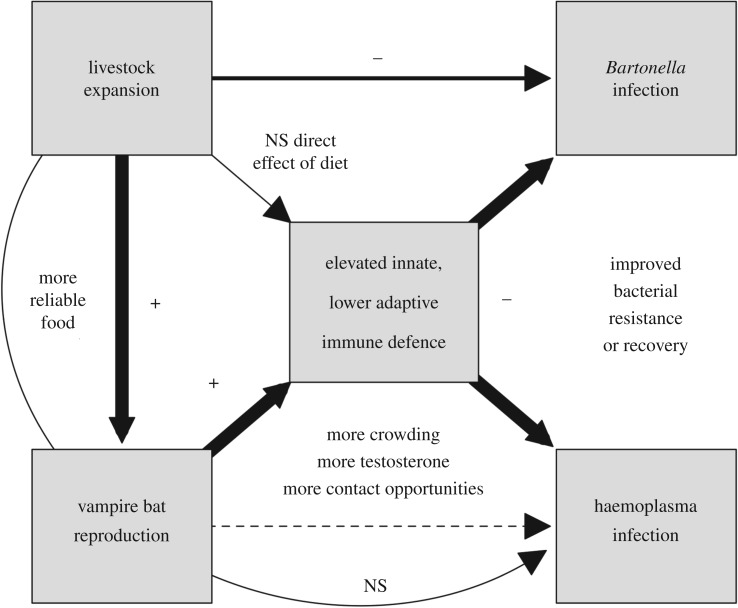
Hypothesized mechanisms affecting bacterial infection in vampire bats in relation to livestock expansion. Signs summarize observed relationships, arrow widths display magnitudes of associations and dashed lines display unobserved mechanisms; NS, not significant.

Whether provisioning amplifies or dampens infection risk depends on how supplemental food affects host demography, immune defence and behaviour, yet studies that simultaneously measure these cross-scale processes and their consequences for infection are rare. Here, we show that provisioning in the form of livestock abundance predicts variation in bat demography, immunity and bacterial infections. Such interactions probably operate through multiple mechanisms (figure 5).

### (a) Provisioning effects on diet and demography

While livestock biomass predicted isotopic indicators of long-term bat diet, our analyses indicate bats in low- and high-livestock habitats feed equally on mammalian livestock and poultry prey; this could suggest that even minor introductions of such prey shift bat feeding towards a domestic animal-dominated diet [24,50]. Given this finding, the positive relationships between livestock biomass and proportions of reproductive and male bats per site could be explained by more abundant feeding opportunities provided by mammalian livestock versus poultry. As vampire bats are highly susceptible to starvation [28], reliable and abundant food provided by livestock could facilitate greater survival and opportunities for reproduction [79]. The higher frequencies of males observed in provisioned sites could be explained by improved maternal condition biasing sex ratios [30,33] or by greater immigration of males into provisioned habitats [30,34].

### (b) Livestock biomass and bat immunity

Bats in high-livestock habitats had a greater proportion of neutrophils in blood, higher BKA and more leucocytes but lower levels of IgG and proportions of lymphocytes. This indicates that abundant livestock might contribute to a shift from adaptive immunity to innate immunity. Livestock biomass was a stronger predictor of this relationship than individual bat diet (figure 5), suggesting an indirect relationship between provisioning and bat immunity and that consistency of feeding on livestock cannot explain these patterns. One explanation could involve differential costs of innate and adaptive immunity. While the adaptive response is typically considered the more costly arm of immunity (particularly in regard to developmental costs), the innate response is inexpensive to develop but can have high energetic and pathological costs to maintain and use [80,81]. As costly defences are predicted to be downregulated when food is limited or other energy demands are high [82], the higher proportions of neutrophils and microbicidal ability for bats in provisioned sites may reflect the ability to allocate more energy towards maintenance of innate defences [15,83]. Alternatively, innate-oriented immunity in provisioned sites may reflect more testosterone production. Higher proportions of reproductive and male bats were captured in such habitats, and reproductive bats displayed innate-oriented immunity. As most reproductive bats were male (145/173), our data may be consistent with prior studies where testosterone enhanced investment in innate immunity [55,84]. Another explanation could be that provisioned bats experience greater chronic stress, which is consistent with higher neutrophil-to-lymphocyte (NL) ratios from these sites [56]. Greater chronic stress could arise if livestock blood is of poor nutritional quality or contaminated [3,48] or if habitat degradation accompanies provisioning [29]. Testing between these hypotheses could be supported by future work quantifying stress hormones (i.e. cortisol) and testosterone in bat tissue with long turnover, such as hair samples. Lastly, innate-oriented immunity in provisioned bats could reflect livestock-rearing practices within highly agricultural sites. While we did not assess whether local livestock were provided with antibiotics or other supplements meant to reduce infection, such practices could directly impair adaptive immunity [85] or promote long-term adaptation to feeding on low-risk prey.

Changes in bat density and associated intraspecific interactions with provisioning could also alter immunity (figure 5). Increased reproductive success and immigration with supplemental feeding could facilitate crowding and food competition [3,86]. For example, tourist-fed southern stingrays displayed more aggressive interactions and higher stress than wild counterparts [87]. While we could not directly quantify bat demographic rates owing to limited recaptures, reproductive activity was more common in high-livestock sites and predicted innate-oriented immune profiles, supporting physiological costs to demographic benefits of provisioning. This relationship is unlikely to be driven by fundamental differences in the immunology of male and female bats combined with the higher frequency of males in provisioned sites, as sex had lower relative importance (figure 2). Another explanation is that innate-oriented immune profiles reflect responses to higher pathogen pressure in provisioned habitats [83,88]. However, while higher NL ratios in provisioned sites support greater acute infection risk [56], our immune PC1 was also negatively loaded by eosinophils, for which declines are consistent with elevated stress hormones [56]. IgG levels were also lower in provisioned sites, inconsistent with overall higher pathogen exposure [61,89]. Elevated markers of innate immunity in provisioned bats are thus more compatible with shifts in energy allocation, male reproductive state and crowding stress.

### (c) Infection correlates of differential immunity

Shifts towards innate immunity associated with higher livestock biomass correlated with lower odds of bat infection with both *Bartonella* and haemoplasmas (figure 5). Although no experimental studies have characterized how bats immunologically respond to either pathogen [90], work on *Bartonella* infection in mice and in cats has identified a role for adaptive immunity (e.g. B and T cells, IFN-γ) in bacterial clearance [91,92]. Given the relationship between our immune PC1 and infection, our data suggest that resistance to or clearance of these bacteria in bats could depend more on innate rather than adaptive immunity. Importantly, *in vitro* studies of humans confirm that complement proteins, which mediate BKA in bat plasma [59], are important in defence against *Bartonella* [93]. Higher odds of infection for bats with more relative investment in lymphocytes and IgG could also indicate adaptive immune responses to bacterial infections, although work to date in bats suggests bacterial challenge stimulates a neutrophil-associated response [94]. Future work employing experimental trials, longitudinal studies and mathematical models will help elucidate if these specific innate immune components (i.e. neutrophils and complement) manifest in bacterial resistance or clearance in bats and their consequences for epidemiology.

### (d) Theory-driven insights into bacterial prevalence

Despite the consistent association between innate-oriented immune profiles and lower odds of bacterial infection, *Bartonella* prevalence showed a stronger negative relationship with livestock biomass. We found that 25% of this association was mediated by the relationship between provisioning and immune profiles, supporting an important role of resource-mediated immune variation for shaping differences in infection [2,10]. For a pathogen probably transmitted via frequency-dependent contact (e.g. bat flies or arthropod vectors [38,39]), *Bartonella* transmission may not increase with the higher bat densities that would be predicted to manifest in provisioned habitats. Without greater pathogen exposure, higher resistance to or recovery from infection should decrease prevalence [10]. Such processes could explain similar patterns of vector-borne disease in response to supplemental food, such as West Nile virus in songbirds [95]. Alternatively, supplemental food could allow bats to spend less time foraging and more time grooming [96], which could lower ectoparasitism and transmission of vector-borne disease [97].

Haemoplasma prevalence also was lower in high-livestock sites but had a quantitatively weaker relationship, which could arise if transmission-enhancing effects of provisioning on bat density and immigration increase contact rates and therefore pathogen transmission [10,23,30]. The negative relationship between relative investment in innate immunity and infection was stronger for haemoplasmas than for *Bartonella*, suggesting that transmission-enhancing processes could be required to offset the expected declines in prevalence [10]. Direct transmission of haemoplasmas via saliva and blood is possible [40,42], particularly given the food-sharing and grooming habits of vampire bats [96,98]. This supports the idea that haemoplasma transmission could increase with provisioning in contexts where bat innate immune response is suppressed.

### (e) Conclusion

Resource provisioning in the form of livestock availability predicts important differences in vampire bat demography and immune defence that could interact to affect infection dynamics in complex ways. Understanding how greater reproduction and relative investment in innate immunity for bats living in livestock-dense habitats affects infection dynamics is complicated owing to multi-scale factors. However, our findings suggest prevalence of vector-borne bacterial pathogens such as *Bartonella* could decline with provisioning, as changes in host demography are less likely to alter transmission but immune defences are heightened by supplemental food. For pathogens that respond more directly to host demographic change, changes in immunity may be insufficient to prevent increases in transmission. An important next step is to disentangle the contribution of resource-altered demography and immunology with a combination of field studies and mechanistic models. This would also be important for predicting how shifts in bat demography and immunity affect viral dynamics. As bats in high-livestock sites also showed lower measures of adaptive immunity (e.g. lymphocytes, IgG) that play key roles in the defence against viruses [99], provisioning might influence bat susceptibility to zoonoses like rabies virus [46,47]. Determining if these field patterns reflect impaired adaptive immunity or reduced viral exposure in livestock-dense habitats, and how these patterns interact with demographic and behavioural processes, will be critical to anticipate how agricultural change will affect risks of pathogen spillover from vampire bats. More broadly, this work shows that considering how resources affect multiple host mechanisms can enhance our understanding of how provisioning affects population-level infection outcomes in wildlife. Given the diversity of ways in which anthropogenic activities subsidize wildlife, this integrative and multi-scale approach in other wildlife systems could enhance our ability to predict and manage emerging disease risks [100].

## Supplementary Material

Supplementary material

## Data accessibility

Data are available in the Dryad Digital Repository: (https://doi.org/10.5061/dryad.904kp) [101].
