# Supplementary material for: Livestock abundance predicts vampire bat demography, immune profiles and bacterial infection risk
Source: Philos Trans R Soc Lond B Biol Sci. 2018 Mar 12;373(1745):20170089. doi: 10.1098/rstb.2017.0089 (PMC5882995; doi:10.1098/rstb.2017.0089)

Livestock abundance predicts vampire bat demography, immune profiles, and bacterial infection risk: Online Supplemental Material

Daniel J. Becker, Gábor Á. Czirják, Dmitriy V. Volokhov, Alexandra B. Bentz, Jorge E. Carrera, Melinda S. Camus, Kristen J. Navara, Vladimir E. Chizhikov, M. Brock Fenton, Nancy B. Simmons, Sergio E. Recuenco, Amy T. Gilbert, Sonia Altizer, Daniel G. Streicker

S1. Site coordinates and livestock density
S2. Stable isotopes of bats and prey
S3. Multivariate analysis of immune function
S4. Livestock biomass and isotopic distance
S5. Sensitivity to holding time

S1. Site coordinates and livestock density

Table S1. Site geography, coordinates, livestock biomass, elevation, and sampling history

Site	Department	Country	Longitude	Latitude	Livestock biomass (kg)[1]		Elevation (m)	Captures	Nights
LR4	Loreto	Peru	-73.204	-4.211	8.839	Low	100	17	7
LR3	Loreto	Peru	-73.218	-4.307	9.881	Low	100	21	7
LR2	Loreto	Peru	-73.199	-4.290	9.967	Low	100	23	8
LR1	Loreto	Peru	-73.303	-4.241	10.251	Low	100	17	7
OW2[2]	Orange Walk	Belize	-88.654	17.753	28.599	High	11	59	11
OW1[2]	Orange Walk	Belize	-88.731	17.816	30.475	High	30	43	9
AM3	Amazonas	Peru	-78.288	-5.201	32.510	High	660	26	4
AM2	Amazonas	Peru	-78.290	-5.212	32.539	High	660	3	2
AM1	Amazonas	Peru	-78.292	-5.212	32.624	High	660	57	13
CA1	Cajamarca	Peru	-78.953	-5.168	36.647	High	1368	103	12

[1]Quarter-root transformed
[2]Based out of the Lamanai Field Research Center

S2. Stable isotopes of bats and prey

Figure S1. Hair isotopes ($\delta^{13}C$ and $\delta^{15}N$) of individual bats and potential prey species per study region (A=Loreto, B=Amazonas and Cajamarca, C=Belize). Circles represent individual bats and are colored by sampling site. Grey triangles correspond to wildlife, black diamonds correspond to mammalian livestock, and black squares correspond to poultry.

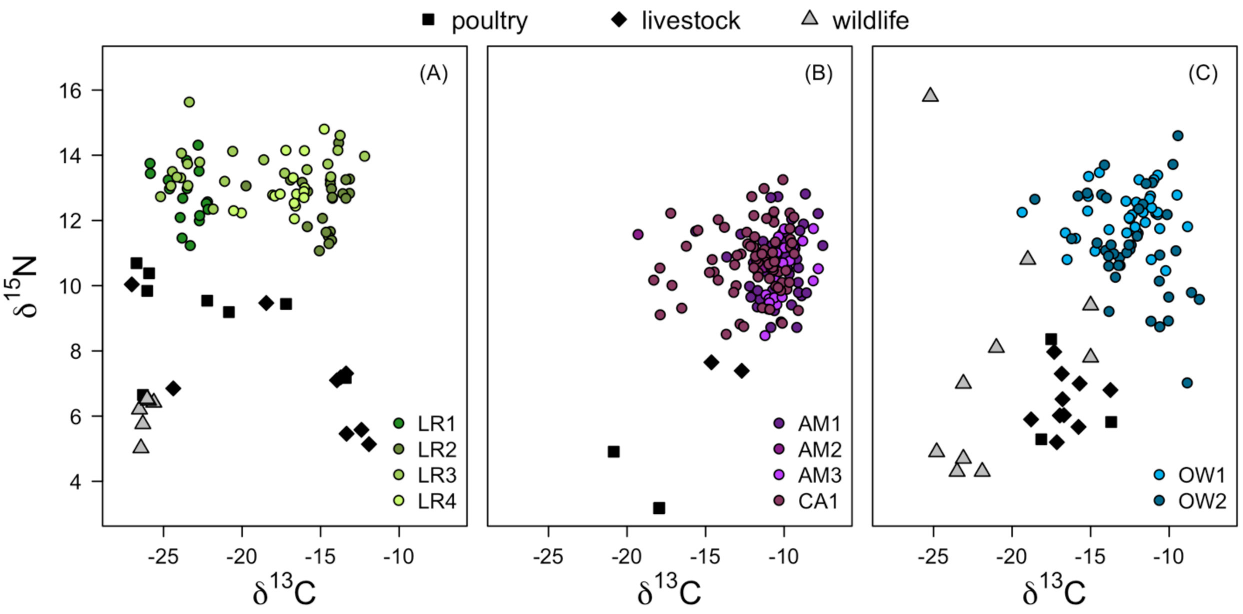

Table S2. Geography, species, and $\delta^{13}C$ and $\delta^{15}N$ values from individual potential bat prey.

Region	Site	Prey species	Prey class	$\delta^{13}C$	$\delta^{15}N$
Amazonas	Puerto Pakuy (near AM1–3)	chicken	poultry	-17.95	3.19
Amazonas	Puerto Pakuy (near AM1–3)	chicken	poultry	-20.85	4.91
Amazonas	Puerto Pakuy (near AM1–3)	chicken	poultry	-17.97	3.17
Cajamarca	CA1	cow	livestock	-14.63	7.65
Cajamarca	CA1	cow	livestock	-12.7	7.39
Belize	OW2	chicken	poultry	-18.14	5.29
Belize	OW2	chicken	poultry	-17.51	8.36
Belize	OW2	chicken	poultry	-13.69	5.82
Belize	OW2	cow	livestock	-16.70	6.03
Belize	OW2	cow	livestock	-16.84	7.30
Belize	OW2	cow	livestock	-18.79	5.90
Belize	OW2	cow	livestock	-15.70	7.00
Belize	OW2	cow	livestock	-16.96	6.02
Belize	OW2	cow	livestock	-17.15	5.20
Belize	OW2	cow	livestock	-16.79	6.52
Belize	OW2	cow	livestock	-13.74	6.80
Belize	OW2	horse	livestock	-15.77	5.67
Belize	OW2	horse	livestock	-17.32	7.97
Belize	OW2	red brocket deer	wild	-23.10	4.70
Belize	OW2	red brocket deer	wild	-23.50	4.30
Belize	OW2	tapir	wild	-24.80	4.90
Belize	Pacbitun (near OW1 & OW2)	peccary	wild	-15.00	7.80
Belize	Pacbitun (near OW1 & OW2)	red brocket deer	wild	-21.90	4.30
Belize	Pacbitun (near OW1 & OW2)	white-tailed deer	wild	-25.20	15.80
Belize	Pacbitun (near OW1 & OW2)	white-tailed deer	wild	-23.10	7.00
Belize	Pacbitun (near OW1 & OW2)	white-tailed deer	wild	-21.00	8.10
Belize	Pacbitun (near OW1 & OW2)	white-tailed deer	wild	-19.00	10.80
Belize	Pacbitun (near OW1 & OW2)	white-tailed deer	wild	-15.00	9.40
Loreto	LR1	chicken	poultry	-26.04	9.84
Loreto	LR1	chicken	poultry	-26.72	10.69
Loreto	LR1	pig	livestock	-24.38	6.85
Loreto	LR2	lowland paca	wild	-26.32	5.76
Loreto	LR2	lowland paca	wild	-26.44	5.02
Loreto	LR3	chicken	poultry	-25.92	10.38
Loreto	LR3	peccary	wild	-26.53	6.21
Loreto	LR3	lowland paca	wild	-25.62	6.42
Loreto	LR4	chicken	poultry	-26.31	6.65
Loreto	LR4	chicken	poultry	-20.84	9.19
Loreto	LR4	pig	livestock	-27.02	10.04
Loreto	Rio Nanay (near Iquitos)	chicken	poultry	-22.22	9.54
Loreto	Rio Nanay (near Iquitos)	chicken	poultry	-17.20	9.44
Loreto	Rio Nanay (near Iquitos)	chicken	poultry	-13.40	7.17
Loreto	Rio Nanay (near Iquitos)	cow	livestock	-13.38	7.31
Loreto	Rio Nanay (near Iquitos)	cow	livestock	-11.92	5.14
Loreto	Rio Nanay (near Iquitos)	cow	livestock	-12.40	5.58
Loreto	Rio Nanay (near Iquitos)	cow	livestock	-13.35	5.46

Loreto	Rio Nanay (near Iquitos)	horse	livestock	-13.72	7.19
Loreto	Rio Nanay (near Iquitos)	horse	livestock	-13.96	7.10
Loreto	Rio Nanay (near Iquitos)	goat	livestock	-18.46	9.47
Loreto	Rio Nanay (near Iquitos)	lowland paca	wild	-25.96	6.48
Loreto	Rio Nanay (near Iquitos)	lowland paca	wild	-26.05	6.53

S3. Multivariate analysis of immune function

Table S3. Pearson correlation coefficients between measures of immune function (*n*=166).

	N	L	M	E	B	TWBC	BKA	IgG
**N**[1]	1							
**L**[2]	–0.98	1						
**M**[3]	–0.36	0.25	1					
**E**[4]	–0.22	0.14	0.29	1				
**B**[5]	–0.02	0.00	0.05	–0.06	1			
**TWBC**[6]	0.15	–0.18	0.16	0.14	–0.08	1		
**BKA**[7]	0.22	–0.21	–0.14	–0.13	0.10	0.13	1	
**IgG**[8]	–0.21	0.18	0.12	0.10	0.07	–0.07	0.09	1

[1]Percent neutrophils from blood smears
[2]Percent lymphocytes from blood smears
[3]Percent monocytes from blood smears
[4]Percent eosinophils from blood smears
[5]Percent basophils from blood smears
[6]Quarter-root transformed total WBC estimates
[7]Percent *E. coli* killed in plasma relative to positive control
[8]Optical density of immunoglobulin G antibody in plasma

Figure S2. Biplot of the first two PCs of eight immune measures. Arrows indicate PC loadings.

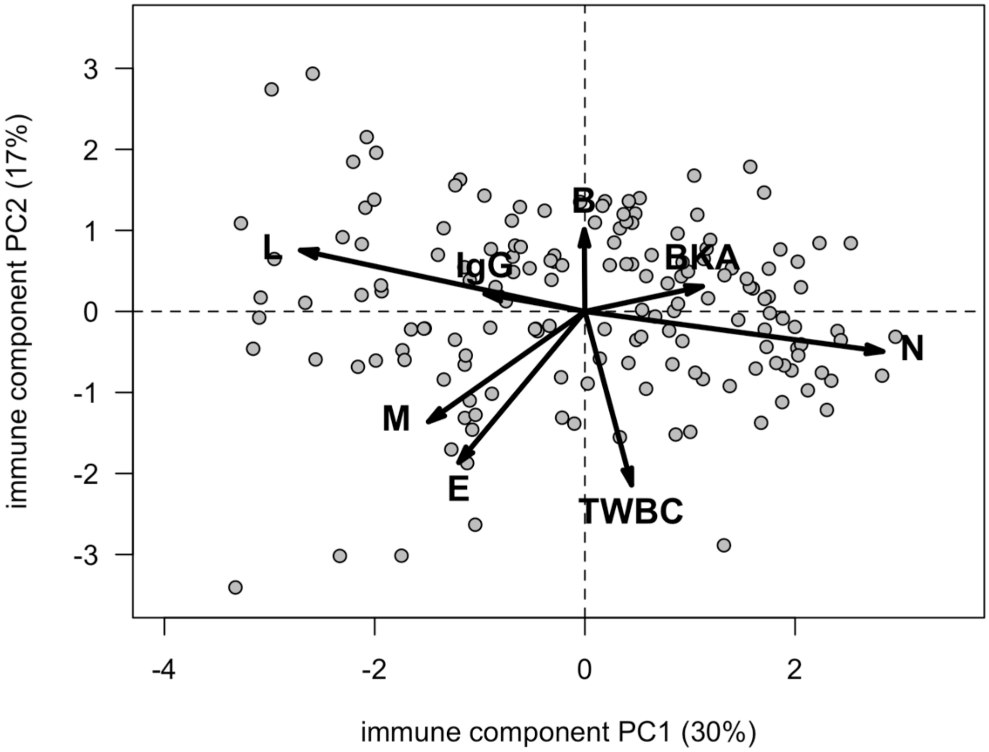

S4. Livestock biomass and isotopic distance

Figure S3. Relationship between livestock biomass and the minimum bat isotopic distance from all livestock and poultry prey per study region. The curve shows the REML fit from the GLMM controlling for inter-annual variation in bat diet.

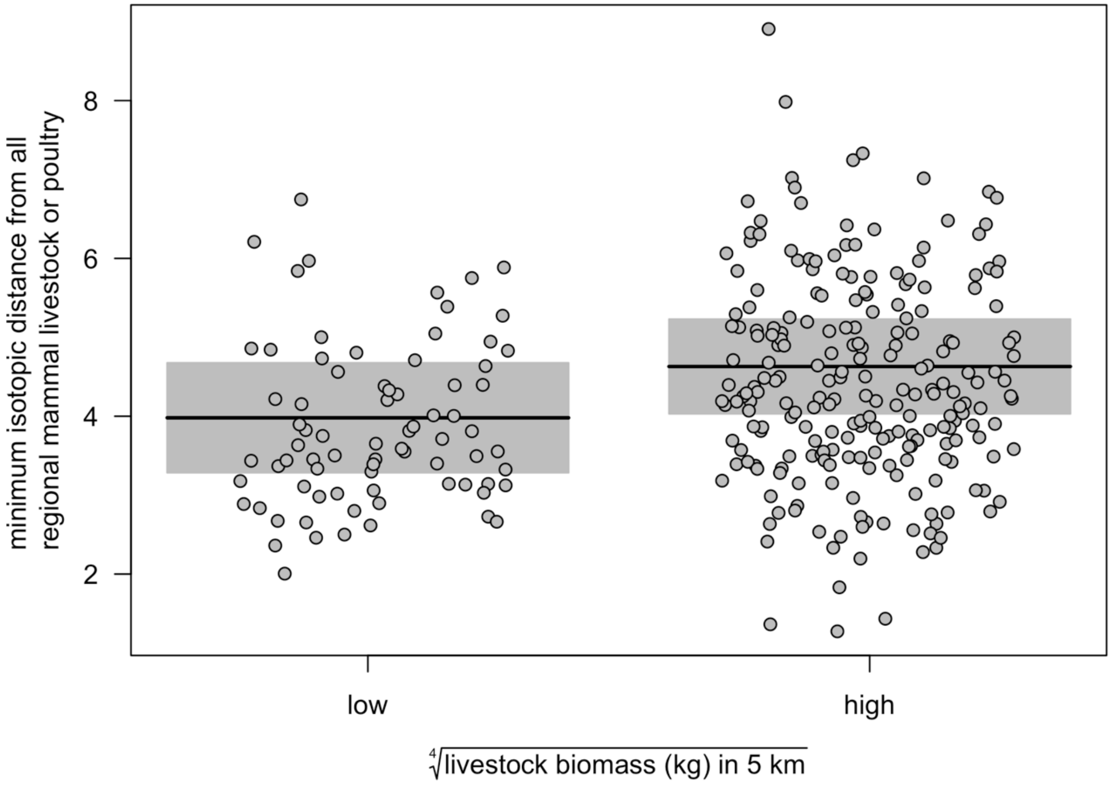

S5. Sensitivity to holding time

Figure S4. Results from model averaging where log holding time was included as a covariate in all GLMMs (A: full dataset; B: restricted to bats held under four hours). The 95% confidence intervals are shown in grey and mean coefficients are shown in black diamonds. The dashed line represents no correlation between covariates and immunity (*β*=0).

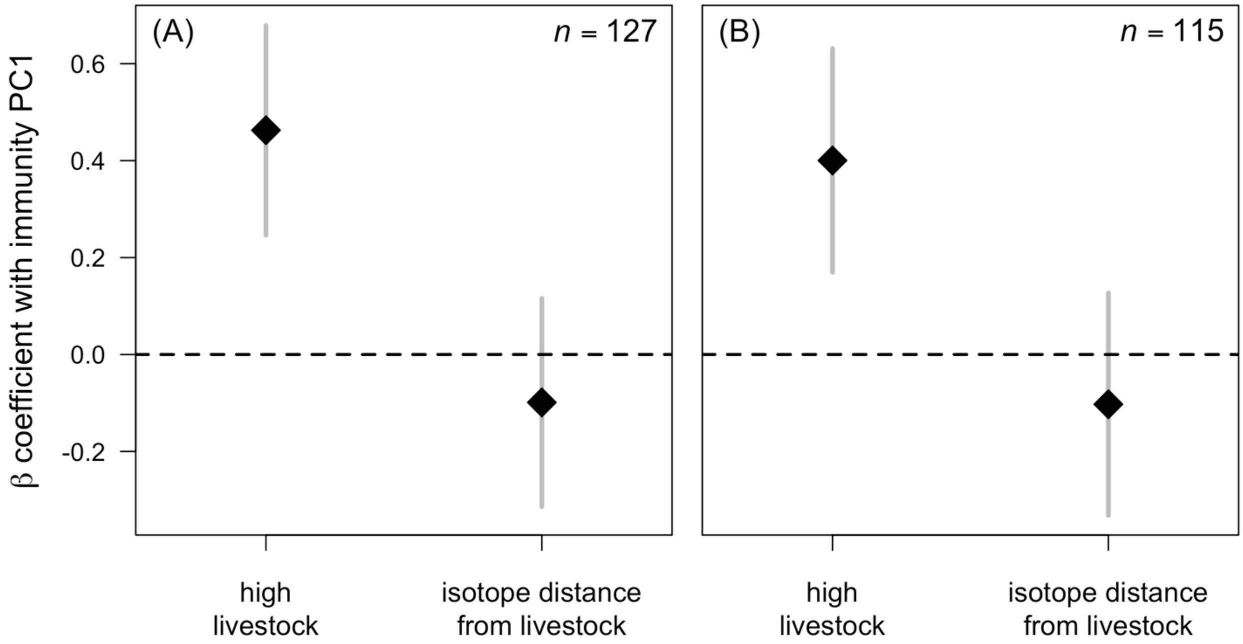

Supplement: Supplementary material [file rstb20170089supp1.pdf]
